# Case report: Sudden onset optic neuritis shortly after SARS-CoV-2 vaccination in an allogeneic hematopoietic stem cell transplant recipient with chronic graft-vs.-host disease

**DOI:** 10.3389/fmed.2023.1177610

**Published:** 2023-06-20

**Authors:** Chiang Chu, Shin-Kuang Jiang, Yi-Ching Shao, Su-Peng Yeh

**Affiliations:** ^1^Division of Hematology and Oncology, Department of Internal Medicine, China Medical University Hospital, Taichung, Taiwan; ^2^Division of Neurology, China Medical University Hospital, Taichung, Taiwan; ^3^Eye Center, China Medical University Hospital, Taichung, Taiwan; ^4^China Medical University, Taichung, Taiwan

**Keywords:** vaccine, graft-vs.-host disease, hematopoietic stem cell transplant, optic neuritis, COVID-19

## Abstract

Hematopoietic stem cell transplantation (HSCT) recipients affected by severe acute respiratory syndrome coronavirus 2 (SARS-CoV-2) have a high mortality rate. The American Society of Transplantation and Cellular Therapy (ASTCT) and the European Society for Blood and Marrow Transplantation (EBMT) recommend vaccination for these vulnerable populations. However, emerging data suggested that vaccination might elicit immunological adverse events, including an exacerbation of graft-vs.-host disease (GVHD). Herein, we report a case of severe optic neuritis developed shortly after AstraZeneca COVID-19 vaccination in an allogeneic HSCT recipient with underlying chronic GVHD. The patient had a headache 5 days after vaccination, and the disease progressed rapidly to complete blindness 17 days after the vaccination. The diagnosis of optic neuritis was well-confirmed by the presence of an anti-myelin oligodendrocyte glycoprotein antibody and the typical features of MRI image and Ophthalmoscopy. Other differential diagnoses, such as infection or leukemia relapse in the central nervous system (CNS), were carefully excluded. A timely high-dose corticosteroid was administered, and her visual acuity improved rapidly. She returned to her baseline status 1 month later. With more than 1 year of follow-up, no optic neuritis or leukemia relapse was observed. In summary, allogeneic transplant recipients can develop severe optic neuritis after vaccination. Optic neuritis can be an exacerbation of GVHD or rarely a sporadic adverse event of vaccination. Furthermore, our experience indicates that a prompt diagnosis and early steroid treatment are vital for a good recovery.

## Introduction

Hematopoietic stem cell transplantation (HSCT) recipients have a higher mortality rate after getting COVID-19. According to a meta-analysis of 14 studies, COVID-19-related mortality was around 4% in the general population before the introduction of vaccination (1). However, the overall survival at 30 days after diagnosis of COVID-19 was 68 and 67% for recipients of allogeneic and autologous HSCT, respectively (2). Vaccination against COVID-19 is thus recommended by major societies such as the American Society of Hematology—American Society of Transplantation and Cellular Therapy (ASH-ASTCT) and the European Society for Blood and Marrow Transplantation (EBMT). Nevertheless, three studies have reported a risk of eliciting or worsening graft-vs.-host disease (GVHD) after the administration of mRNA vaccines (3–5). The safety data of AstraZeneca (AZ) adenoviral vaccine on the HSCT recipients are sparse. Herein, we introduce a rare case of anti-myelin oligodendrocyte glycoprotein (anti-MOG) antibody-positive optic neuritis developed shortly after AZ vaccination in a recipient of allogeneic HSCT with underlying chronic GVHD (cGVHD).

## Case description

A 60-year-old female presented to the clinic with a history of acute onset of visual loss 17 days after receiving the first dose of the AstraZeneca (AZ) COVID-19 vaccine. She underwent a matched unrelated donor peripheral blood stem cell transplant for AML in 2009. Mild cGVHD involving only the lacrimal gland (sicca syndrome) developed after the transplant, and she needs a regular artificial tear for symptom control. Neither topical steroids nor systemic immunosuppressive agents were used. The patient received the first dose of the AZ vaccine during the COVID-19 pandemic (the Day of vaccination is defined as Day 0). She suffered from intermittent headaches since Day 5, followed by bilateral periorbital pain on Day 14. Blurred vision of left eye developed on Day 15 and rapidly progressed to right eye on Day 17 with total blindness of left eye. Outside the ocular problem, the patient did not have other neurological symptoms, such as focal weakness or sensory deficit. There were also no fever, joint pain, and other symptoms suggesting infectious or rheumatological diseases.

**Figure 1:**
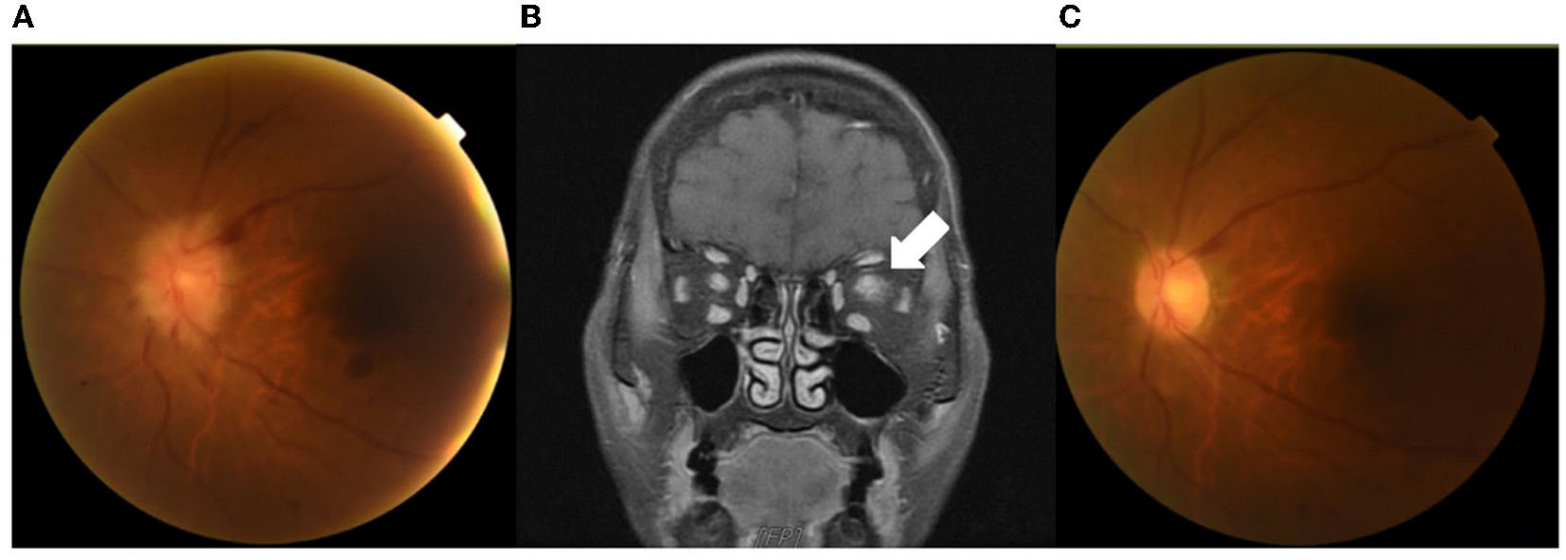
Images of contrast MRI and ophthalmoscopy. An ophthalmoscopic examination on Day 18 showed left optic disk papilledema with retinal hemorrhage **(A)**. Brain MRI after gadolinium administration on the same day showed swelling of the left optic nerve with increased enhancement (arrow), compatible with optic neuritis **(B)**. An ophthalmoscopic examination on Day 45 showed the resolution of papilledema and retinal hemorrhage **(C)**.

The nasopharyngeal swab PCR for COVID-19 was negative. Ophthalmoscopic examination on Day 18 disclosed left optic disk papilledema with retinal hemorrhage (Figure 1A). Contrast brain MRI revealed swelling of the left optic nerve with increased enhancement, compatible with optic neuritis (Figure 1B). A cerebrospinal fluid (CSF) study showed no pleocytosis (WBC 5 cells/μL). The IgG index was 0.64 (normal range 0.3–0.7), and the micro-albumin was 27.0 mg/dL (normal range 10–30 mg/dL). Microbiological studies of CSF using FilmArray Meningitis/Encephalitis panel (BioFire Diagnostics, Salt Lake City, UT), which detects Escherichia coli, Haemophilus influenzae, Listeria monocytogenes, Neisseria meningitidis, Streptococcus agalactia and streptococcus pneumoniae, Cytomegalovirus, Enterovirus, Herpes simplex virus (HSV)-1, HSV-2, Human herpesvirus-6, Human parechovirus, Varicella zoster virus, and Cryptococcus, were all negative. Bone marrow biopsy showed no leukemia relapse. Whole-body positron emission tomography/computed tomography scan revealed a negative finding. Anti-aquaporin-4 antibody was negative, and the anti-MOG antibody (cell-based immunofluorescence test) was positive.

The patient received pulse therapy with methylprednisolone 250 mg daily from Day 19 to 21, then switched to oral prednisolone 1 mg/kg from Day 22. Her visual acuity improved rapidly and has almost returned to her baseline status on Day 28. Repeated ophthalmoscopic examination on Day 42 shows no papillary or retinal edema. The prednisolone was tapered off gradually over the next 4 months. We have followed the patient for over a year, and no optic neuritis or leukemia relapse has occurred. The patient reported that the ocular condition is now the same as before the vaccination. However, using artificial tears of roughly the same frequency remains needed to control dry eyes.

## Discussion

Optic neuritis is a rare immune-mediated neurological disturbance. While ocular involvement is commonly seen in patients with cGVHD, the process is most often limited to the anterior eye segment. Few cases of cGVHD with the presentation of optic neuritis have been reported, but none were associated with vaccination (Table 1) (6–9). Besides, our case is the first report of documented anti-MOG positivity in this setting, which further supports the attacks of immune-mediated demyelination targeting optic nerves.

**Table 1:** Cases of cGVHD with the presentation of optic neuritis reported in the literature.

**Reports (reference)**	**Age/Sex**	**Diagnosis**	**Organs affected by cGVHD**	**CSF**		**Serology test**	**Treatments**	**Follow-up**	**Outcome**
				**WBC**	**Protein**				
Diamanti et al. (6)	51 F	AML	Hashimoto's thyroiditis	39 lym./μL	No data	AQP4 positive NMOSD	Pulse steroid Plasma exchange Rituximab	8 months	No recovery
Moesen and Kidd (7)	21 F	HD	Bronchitis obliterans	No cell	230 mg/dl	NA	Pulse steroid Mycophenolate	6 weeks	Partial recovery
Matsuo et al. (8)	22 F	MDS	Hepatitis Lichen planus	3 Cells/μL	20 mg/dl	NA	Cyclosporine A	2 years	Partial recovery
Ooi et al. (9)	34 M	CML	Grade I Acute GVHD	24 lym./μL	77 mg/dl	NA	Pulse steroid	6 months	Recovery
This case	60 F	AML	Sicca syndrome	5 Cells/μL	42.8 mg/dL	Anti-MOG positive Optic neuritis	Pulse steroid	14 months	Recovery

The safety issue of vaccination for patients with active GVHD is unclear because most clinical trials likely exclude them. According to a retrospective cohort study that enrolled 34 patients diagnosed with cGVHD and received two doses of mRNA SARS-CoV-2 vaccine during the COVID-19 pandemic, nine (26.5%) experienced worsening of their cGVHD symptoms after vaccination (10). All these nine patients received the Pfizer mRNA vaccine, and the most common organs involved are the skin, mouth, and eyes. Ali et al. also reported a worsening of cGVHD in 3.5% and new GVHD in 9.7% of allogeneic HSCT recipients who received Pfizer (BNT162b2) or Moderna (mRNA-1273) vaccines (10). Our case is unique in its provoked by the vector-based AZ vaccine and the unusual site of immune attack. The exact mechanism of vaccination-associated cGVHD remains unknown, although significantly higher cytokine (Interleukin-18, Angiopoietin-2, Interferon-gamma) were observed in patients with suspected cGVHD aggravation after vaccination (11). On the other hand, sporadic cases of anti-MOG positive optic neuritis after the AZ COVID-19 vaccine had been reported in non-transplant patients. However, most of these cases showed multiple site involvements or combined with myelitis or extensive encephalomyelitis rather than isolated optic neuritis (12). Whether the optic neuritis seen in this patient is a GVHD aggravation or a new, vaccination-associated event is not very critical because the treatment for both conditions is likely the same. A prompt diagnosis and early corticosteroid treatment are even more important. In the current case, we used a high-dose steroid as the initial treatment, which aligns with the recommendation for treating idiopathic optic neuritis with significant visual loss (13). The patient got a robust and sustained response, and there was no recurrence of neuritis after discontinuation of steroid.

In conclusion, although SARS-CoV-2 vaccination is generally considered safe and recommended for allogeneic HSCT patients, severe optic neuritis could develop due to GVHD exacerbation or vaccination itself. Careful monitoring after vaccination remains warranted, especially in patients with active GVHD. A quick diagnosis and early administration of high dose steroid can rapidly restore visual acuity.

## Data availability statement

The original contributions presented in the study are included in the article/supplementary material, further inquiries can be directed to the corresponding author.

## Ethics statement

Written informed consent was obtained from the participant/patient(s) for the publication of this case report.

## Author contributions

S-KJ and Y-CS conducted the clinical consultation and diagnostics. CC was involved in patient care and wrote the first draft. S-PY was involved in patient care and revised the manuscript. All authors have read and approved the submitted version.
